# Multiple Cross Displacement Amplification Coupled With Nanoparticles-Based Lateral Flow Biosensor for Detection of *Staphylococcus aureus* and Identification of Methicillin-Resistant *S. aureus*

**DOI:** 10.3389/fmicb.2018.00907

**Published:** 2018-05-09

**Authors:** Yi Wang, Weiqiang Yan, Shanshan Fu, Shoukui Hu, Yan Wang, Jianguo Xu, Changyun Ye

**Affiliations:** ^1^State Key Laboratory for Infectious Disease Prevention and Control, National Institute for Communicable Disease Control and Prevention, Collaborative Innovation Center for Diagnosis and Treatment of Infectious Diseases, Chinese Center for Disease Control and Prevention, Beijing, China; ^2^Department of Laboratory, Zunyi Maternal and Child Health Hospital, Guizhou, China; ^3^Department of Clinical Laboratory, Peking University Shougang Hospital, Beijing, China

**Keywords:** *S. aureus*, MRSA, MSSA, MCDA, LFB

## Abstract

*Staphylococcus aureus* (*S. aureus*), including methicillin-resistant *S. aureus* (MRSA), is one of the most important human pathogens, which is responsible for bacteremia, soft-tissue infections, and food poisoning. Hence, multiple cross displacement amplification (MCDA) is employed to detect all *S. aureus* strains, and differentiates MRSA from methicillin-sensitive *S. aureus*. Multiplex MCDA (m-MCDA), which targets the *nuc* gene (*S. aureus*-specific gene) and *mecA* gene (encoding penicillin-binding protein-2′), could detect *S. aureus* strains and identify MRSA within 85 min. Detection of the m-MCDA products is achieved using disposable lateral flow biosensors. A total of 58 strains, including various species of Gram-positive and Gram-negative strains, are used for evaluating and optimizing m-MCDA assays. The optimal amplification condition is found to be 63°C for 40 min, with detection limits at 100 fg DNA/reaction for *nuc* and *mecA* genes in the pure cultures, and 10 CFU/tube for *nuc* and *mecA* genes in the blood samples. The analytical specificity of m-MCDA assay is of 100%, and no cross-reactions to non-*S. aureus* strains are produced according to the specificity testing. Particularly, two additional components, including AUDG enzyme and dUTP, are added into the m-MCDA amplification mixtures, which are used for eliminating the unwanted results arising from carryover contamination. Thus, the m-MCDA technique appears to be a simple, rapid, sensitive, and reliable assay to detect all *S. aureus* strains, and identify MRSA infection for appropriate antibiotic therapy.

## Introduction

*Staphylococcus aureus* (*S. aureus*) is a commensal organism, and approximately of 30% of human population is colonized with the bacterium (Tong et al., 2015). Simultaneously, *S. aureus* also is a human pathogen, and it has the ability to cause serious infections in human (Hennekinne et al., 2012). The pathogen is one of the common causes of food-borne disease, and also is one of the most important bacteria in hospital-acquired and community-acquired infections related to high mortality (Stryjewski and Corey, 2014). Since the introduction of methicillin, the methicillin-resistant *S. aureus* (MRSA) has occurred and spread worldwide, and the infections caused by MRSA have become a serious problem and were associated with significant mortality and morbidity, especially in patients with bacteremia (Jacob and DiazGranados, 2013). In developing countries, the MRSA has emerged in over 60% of isolated *S. aureus*, and the occurrence of methicillin resistance in *S. aureus* causing infection has exceeded 49% in the United States hospitals and increased steadily (Jernigan, 2004; Misawa et al., 2007). Herein, advanced assays are needed for rapid detection and accurate differentiation of methicillin-susceptible *S. aureus* (MSSA) and MRSA infections to ensure optimal therapy and management of patients.

Conventional MSSA and MRSA detection methods, including growth-based techniques, colony morphology, and micro-dilution resistance examinations, are laborious and time-consuming, and even with a positive blood culture (Law et al., 2014). In particular, these procedures take about 2 days to identify MRSA after a positive result (Chen et al., 2017). For this reason, glycopeptide treatment is implemented as an empirical therapy in patients with suspected MRSA infection until the antibiotic susceptibility examination results are provided. As a result, the empirical application of glycopeptides further increased the pressure for the selection of vancomycin resistance (Paule et al., 2005). Thus, the rapid identification and reliable differentiation of MSSA and MRSA could accelerate the diagnosis of *S. aureus* infection and reduce the level of empirical use of antibiotic.

In recent years, many clinical laboratories have established and used the molecular techniques for rapidly detecting and determining the antimicrobial susceptibility patterns of bacterial isolates from clinical samples (Carroll, 2008). Polymerase chain reaction (PCR)-based molecular techniques, including conventional PCR and real-time PCR, have been employed for *S. aureus* detection and determination of methicillin resistance in clinical samples (Tan et al., 2001; Hallin et al., 2003). However, PCR-based approaches had limitations in simplicity and rapidity, due to long genomic template extraction protocols or the requirement for individual PCRs to provide all necessary products. Moreover, several methods using the LightCycler system (Roche Diagnostics, GmbH, Mannheim, Germany) were extremely expensive (Shrestha et al., 2002; Wellinghausen et al., 2004). Hence, a simple, timely, cost-effective and highly efficient assay for diagnosing *S. aureus* and detecting their methicillin resistance should be developed.

In order to achieve more such effective diagnostic tools, we employ a novel nucleic acid amplification technique, multiple cross displacement amplification (MCDA) (Wang et al., 2015), to detect *S. aures* and identify methicillin resistance. MCDA assay is able to amplify nucleic acid sequences with high efficiency and sensitivity using simple reaction instruments under isothermal conditions (Wang et al., 2018a). In the MCDA system, a set of 10 primers, including 2 displacement primers (F1 and F2), 2 cross primers (CP1 and CP2), and 6 amplification primers (C1, D1, R1, C2, D2, and R2), were specially designed on the target sequences, thus MCDA assays possessed high selectivity for target sequence detection. More recently, to further simplify diagnostic tools and achieve multiplex MCDA (m-MCDA) detection, the conventional MCDA technique combined with nanoparticle-based lateral flow biosensor (MCDA-LFB) are developed, promising rapid, simple, multiplex, and visual detection of target sequences in clinical diagnostics and serving as a point-of-care device (Wang et al., 2017a). Moreover, MCDA-LFB technique is integrated with antarctic thermal sensitive uracil-DNA-glycosylase (AUDG) digestion to remove the carryover contamination, thus the false-positive results arising from contaminants are eliminated (Wang et al., 2017a, 2018a,b). As a potentially valuable tool for the rapid diagnosis of pathogen infection, we report on a method for detection of MSSA and MRSA by MCDA-LFB assay and attempt to investigate the potential clinical impact of the more rapid provision of examination results.

## Materials and Methods

### Reagents and Apparatus

Rabbit anti-fluorescein antibody (anti-FITC), sheep anti-digoxigenin antibody (anti-Dig), and biotinylated bovine serum albumin (biotin-BSA) were obtained from Abcam Co., Ltd. (Shanghai, China). Dye (Crimson red) streptavidin-coated polymer nanoparticles (SA-DNPs; 129 nm, 10 mg mL^-1^, 100 mM borate, pH 8.5 with 0.1% BSA, 0.05% Tween 20, and 10 mM EDTA) were obtained from Bangs Laboratories, Inc. (Fishers, Indiana, United States). Biotin-14-dCTP was obtained from Thermo Scientific. Co., Ltd. (Shanghai, China). Antarctic thermal sensitive uracil-DNA-glycosylase (AUDG), dATP, dTTP, dCTP, and dGTP was obtained from New England Biolabs, Inc. (Beijing, China). Isothermal Amplification kits, visual detection reagent (Malachite Green, MG) and dUTP were obtained from BeiJing-HaiTaiZhengYuan Technology Co., Ltd. (Beijing, China). The backing card, sample pad, conjugate pad, nitrocellulose membrane (NC), and absorbent pad were obtained from the Jieyi Biotechnology Co., Ltd. (Shanghai, China). The DNA extraction kits (QIAamp DNA Mini Kits; Qiagen, Hilden, Germany) were obtained from Qiagen (Beijing, China).

### Primer Design

**FIGURE 1:**
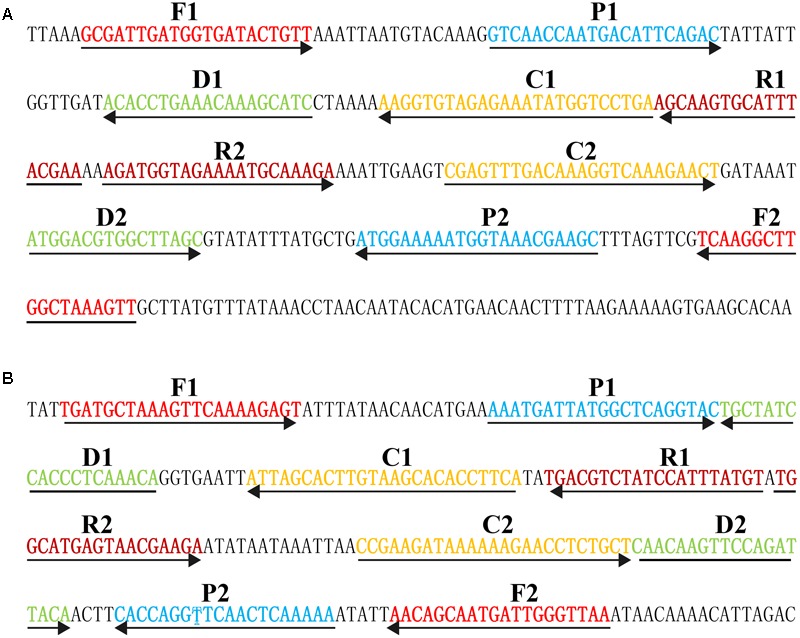
Sequence and location of *nuc* **(A)** and *mecA* **(B)** genes used to design multiple cross displacement amplification primers. The nucleotide sequences of the sense strand of *nuc* and *mecA* are listed. Right arrows and left arrows indicate sense and complementary sequences that are used.

Based on the mechanism of MCDA, two sets of MCDA primers used for *S. aureus* and MRSA detection were designed targeting *nuc* (GenBank accession EF529597) and *mecA* (GenBank Accession No. X52593) genes, respectively. The details of MCDA primers used in the report were shown in **Figure 1** and **Table 1**. All MCDA primers were commercially synthesized by Ruibo-Xingke Biotech Co., Ltd. (Beijing, China).

**Table 1:** The primers used in the current report.

Primers name^a^	Sequences and modifications^b^	Length^c^	Genes
nuc-F1	5′-GCGATTGATGGTGATACTGTT-3′	21 nt	*nuc*
nuc-F2	5′-AACTTTAGCCAAGCCTTGA-3′	19nt	
nuc-CP1	5′-TCAGGACCATATTTCTCTACACC TTGTCAACCAATGACATTCAGAC-3′	46 mer	
nuc-CP2	5′-CGAGTTTGACAAAGGTCAAAG AACTGCTTCGTTTACCATTTTTCCAT-3′	47 mer	
nuc-C1	5′-TCAGGACCATATTTCTCTACACCTT-3′	25 nt	
nuc-C1^∗^	5′-FITC-TCAGGACCATATTTC TCTACACCTT-3′	25 nt	
nuc-C2	5′-CGAGTTTGACAAAGGTCAAAGAACT-3′	25 nt	
nuc-D1	5′-GATGCTTTGTTTCAGGTGT-3′	19 nt	
nuc-D2	5′-ATGGACGTGGCTTAGC-3′	16 nt	
nuc-R1	5′-TTCGTAAATGCACTTGCT-3′	18 nt	
nuc-R2	5′-AGATGGTAGAAAATGCAAAGA-3′	21 nt	
mecA-F1	5′-TGATGCTAAAGTTCAAAAGAGT-3′	22 nt	*mecA*
mecA-F2	5′-TTAACCCAATCATTGCTGTT-3′	20 nt	
mecA-CP1	5′-TGAAGGTGTGCTTACAAGTGCTAATAA ATGATTATGGCTCAGGTAC-3′	46 mer	
mecA-CP2	5′-CCGAAGATAAAAAAGAACCTCTGCT TTTTTGAGTTGAACCTGGTG-3′	45 mer	
mecA-C1	5′-TGAAGGTGTGCTTACAAGTGCTAAT-3′	25 nt	
mecA-C1^∗^	5′-Dig-TGAAGGTGTGCTTACA AGTGCTAAT-3′	25 nt	
mecA-C2	5′-CCGAAGATAAAAAAGAACCT CTGCT-3′	25 nt	
mecA-D1	5′-TGTTTGAGGGTGGATAGCA-3′	19 nt	
mecA-D2	5′-CAACAAGTTCCAGATTACA-3′	19nt	
mecA-R1	5′-ACATAAATGGATAGACGTCA-3′	20 nt	
mecA-R2	5′-TGGCATGAGTAACGAAGA-3′	18 nt	

*^*a*^nuc, *nuc* gene; mecA, *mecA* gene; nuc-C1^∗^, 5′-labeled with FITC when used in MCDA-LFB assay; mecA-C1^∗^, 5′-labeled with Dig when used in MCDA-LFB assay; ^*b*^FITC, fluorescein isothiocyanate; Dig, digoxigenin; ^*c*^ mer, monomeric unit; nt, nucleitide.*

### Bacterial Strains and Genomic DNA Preparation

Of the total of 58 bacterial strains, including two *S. aureus* reference strains (ATCC 43300 and ATCC 25923), 14 methicillin-resistant *S. aureus*, 12 methicillin-sensitive *S. aureus* (MSSA), and 30 non-*S. aureus* strains, were used in the current study (**Table 2**). The reference strain of *S. aureus* (ATCC 43300) was used for the optimization of MCDA assay. According to the manufacture’s instructions, the DNA templates were extracted using DNA extraction kits (QIAamp DNA Mini Kits, Hilden, Germany) and were quantified using ultraviolet spectrophotometer (Nano drop ND-1000, Calibre, Beijing, China) at A260/280. DNA templates of *S. aureus* ATCC 43300 were serially diluted (10 ng, 10 pg, 1 pg, 100 fg, 10 fg, 1 fg, and 100 ag per microliter) and a volume of 1 μl of each dilution was added into the MCDA reactions.

**Table 2:** Bacterial strains used in the current study.

Bacteria^a^	Strain no. (source of strains)^c^	No. of strains	m-MCDA result^d^	
			*Nuc*	*mecA*
***S. aureus species***				
*S. aureus* (MRSA)	ATCC 43300	1	P	P
*S. aureus* (MRSA)^b^	Isolated strains (SG)	14	P	P
*S. aureus* (MSSA)	ATCC 25923	1	P	N
*S. aureus* (MSSA)	Isolated strains (ICDC)	12	P	N
***Non-S. aureus species***				
*Vibrio cholerae*	ATCC14035	1	N	N
*Campylobacter jejuni*	ATCC33291	1	N	N
*Pseudomonas aeruginosa*	Isolated strains (ICDC)	1	N	N
*Staphylococcus epidermidis*	Isolated strains (ICDC)	1	N	N
*Vibrio alginolyticus*	Isolated strains (ICDC)	1	N	N
*Plesiomonas shigelloides*	Isolated strains (ICDC)	1	N	N
*Aeromonas hydrophila*	Isolated strains (ICDC)	1	N	N
*Enterohemorrhagic E. coli*	EDL933 (ICDC)	1	N	N
*Enteropathogenic E. coli*	Isolated strains (ICDC)	1	N	N
*Enterotoxigenic E. coli*	Isolated strains (ICDC)	1	N	N
*Enteroaggregative E. coli*	Isolated strains (ICDC)	1	N	N
*Enteroinvasive E. coli*	Isolated strains (ICDC)	1	N	N
*Shigella dysenteriae*	Isolated strains (ICDC)	1	N	N
*Shigella boydii*	Isolated strains (ICDC)	1	N	N
*Shigella flexneria*	Isolated strains (ICDC)	1	N	N
*Shigella sonneri*	Isolated strains (ICDC)	1	N	N
*Salmonella*	Isolated strains (ICDC)	1	N	N
*Enterococcus faecalis*	ATCC35667	1	N	N
*Enterococcus faecium*	Isolated strains (ICDC)	1	N	N
*Listeria monocytogenes*	ATCC-EGD-e	1	N	N
*Listeria ivanovii*	ATCCBAA-678	1	N	N
*Listeria grayi*	ATCC25402	1	N	N
*Listeria innocua*	Isolated strains (ICDC)	1	N	N
*Listeria welshimeri*	Isolated strains (ICDC)	1	N	N
*Listeria seeligeri*	Isolated strains (ICDC)	1	N	N
*Yersinia enterocolitica*	ATCC23715	1	N	N
*Enterobacter cloacae*	Isolated strains (ICDC)	1	N	N
*Streptococcus pneumonia*	ATCC700674	1	N	N
*Bacillus cereus*	Isolated strains (ICDC)	1	N	N
*Klebsiella pneumoniae*	Isolated strains (ICDC)	1	N	N

*^*a*^MRSA, methicillin-resistant *S. aureus*; MSSA, methicillin-sensitive *S. aureus* (MRSA). ^*b*^These genomic templates were kindly provided by Prof. SH, Department of clinical laboratory, Peking University Shougang Hospital, Shijingshan, Beijing, China. ^*c*^ATCC, American Type Culture Collection; SG, Peking University Shougang Hospital; ICDC, National Institute for Communicable Disease Control Disease Control and Prevention, Chinese Center for Disease Control and Prevention. ^*d*^P, positive; N, negative. Only *S. aureus* strains could be detected by the m-MCDA technique, indicating the extremely high selectivity of the method. The MRSA strains and MSSA strains could be differentiated using LFB detection.*

### Preparation and Operation of Lateral Flow Biosensor (LFB)

The LFB was prepared according to previous studies and used for visual detection of MCDA products (Wang et al., 2017a,c). In brief, the LFB platform contains an immersion pad, a conjugate pad, two test lines (TL I and TL II), a control line (CL), and an absorbent pad. Dye (Crimson red) SA-DNPs were gathered in the conjugate pad, and anti-FITC, anti-Dig, and biotin-BSA were affixed at the TL I, TL II, and CL, respectively. First, a 0.4 μl aliquot of MCDA products, including FITC and biotin-labeled MCDA amplicons, Dig and biotin-labeled MCDA amplicons, was loaded into the immersion pad. Next, a 70 μl aliquot of running buffer also was added into the immersion pad, thus the capillary flow could transfer MCDA products and SA-DNPs from the conjugate pad to TL I, TL II, and CL. At the conjugated pad, the biotin-labeled MCDA amplicons could form a complex with SA-DNPs via biotin-streptavidin interactions. The biotin/MCDA complexes were captured at the TL I by interaction between anti-FITC and FITC, and at the TL II by interaction between anti-Dig and Dig, whereas the SA-DNPs that did not form complexes were immobilized at the CL by interaction between biotin and streptavidin. As a result, FITC/MCDA/SA-DNPs complexes, Dig/MCDA/SA-DNPs complexes, and non-complexed SA-DNPs were indicated by crimson red lines at the TL I, TL II, and CL, respectively. The colorimetric bands were easily visible to the naked within 2 min.

### MCDA Reactions

The *Nuc*-MCDA reactions is performed in a one-step reaction in a 25-μl mixture containing 2.5 μl 10 X of the supplied buffer, 0.4 μM each of displacement primers nuc-F1 and nuc-F2, 0.8 μM each of amplification primers nuc-C1^∗^, nuc-C2, nuc-R1, nuc-R2, nuc-D1, and nuc-D2, 1.6 μM each of cross primers nuc-CP1 and nuc-CP2, 0.8 M betaine (Sigma-Aldrich), 1.4 mM dATP, 1.0 mM dCTP, 0.4 mM biotin-14-dCTP, 1.4 mM dGTP, 1.4 mM dUTP, 1 μl (8 U) of *Bst* 2.0 DNA polymerase, 0.3 μl (0.3 U) of AUDG, and 1 μl DNA template.

The *MecA*-MCDA reactions is performed in a one-step reaction in a 25-μl mixture containing 2.5 μl 10 X of the supplied buffer, 0.4 μM each of displacement primers mecA-F1 and mecA-F2, 0.8 μM each of amplification primers mecA-C1^∗^, mecA-C2, mecA-R1, mecA-R2, mecA-D1, and mecA-D2, 1.6 μM each of cross primers mecA-CP1 and mecA-CP2, 0.8 M betaine (Sigma-Aldrich), 1.4 mM dATP, 1.0 mM dCTP, 0.4 mM biotin-14-dCTP, 1.4 mM dGTP, 1.4 mM dUTP, 1 μl (8 U) of *Bst* 2.0 DNA polymerase, 0.3 μl (0.3 U) of AUDG, and 1 μl DNA template.

The m-MCDA reactions are performed in a one-step reaction in a 25-μl mixture containing 2.5 μl 10 X of the supplied buffer, 0.4 μM each of displacement primers nuc-F1 and nuc-F2, 0.4 μM each of amplification primers nuc-C1^∗^, nuc-C2, nuc-R1, nuc-R2, nuc-D1, and nuc-D2, 0.8 μM each of cross primers nuc-CP1 and nuc-CP2, 0.4 μM each of displacement primers mecA-F1 and mecA-F2, 0.4 μM each of amplification primers mecA-C1^∗^, mecA-C2, mecA-R1, mecA-R2, mecA-D1, and mecA-D2, 0.8 μM each of cross primers mecA-CP1 and mecA-CP2, 0.8 M betaine (Sigma-Aldrich), 1.4 mM dATP, 1.0 mM dCTP, 0.4 mM biotin-14-dCTP, 1.4 mM dGTP, 1.4 mM dUTP, 1 μl (8 U) of *Bst* 2.0 DNA polymerase, 0.3 μl (0.3 U) of AUDG, and 1 μl DNA template.

Monitoring methods, including colorimetric indicator (MG), real-time turbidity (LA-320C), and LFB detection, are used for the verifying and confirming the *nuc*-MCDA, *mecA*-MCDA, and m-MCDA products. The strategy of visualizing MCDA products on LFB was adapted from previous reports (Wang et al., 2017a).

Then, we tested the optimal temperatures of two sets of MCDA primers (nuc-MCDA primers and mecA-MCDA primers) during the amplification stage. Reaction temperatures ranging from 61 to 66°C at 1°C intervals were compared and reaction mixtures with 1 μl of DNA template of *L. monocytogenes* (ATCC 19114) and *S. pneumonia* (ATCC700674) were used as negative controls (NCs), and 1 μl of double distilled water (DW) were used as a blank control (BC).

### Sensitivity of MCDA Assays

The serial dilution of the ATCC 43300 to cover the range of 10 ng to 100 aq was prepared, and 1 μl of genomic DNA was added into the amplification mixtures. Singlex (nuc-MCDA and mecA-MCDA) and m-MCDA reactions were carried out as described above to examine the LoD (limit of detection). The LoD of singlex and multiplex reactions was confirmed as the last dilution of each positive test.

Then, optimal duration of time required for the m-MCDA method during the reaction stage was tested. Four amplification times, including 20, 30, 40, and 50 min, were compared at the optimal amplification temperature, and the m-MCDA products were detected using LFB.

### Simulating Carryover Contamination

The m-MCDA products obtained from 10 pg/μl in absence of AUDG enzyme were quantitated using ultraviolet spectrophotometer (NanoDrop ND-1000, Calibre, Beijing, China). Then, the m-MCDA products were applied for making serial dilution from 1 × 10^-13^ to 1 × 10^-20^ g μL^-1^. A volume of 1 μl of each dilution was added into m-MCDA reaction mixtures as templates, which were used as the source of simulating carryover contaminants.

### Elimination of Carryover Contamination by AUDG Enzyme

In the current report, we evaluated the ability of AUDG enzyme to prevent the false-positive amplifications due to carryover contaminants in detecting target pathogens. The m-MCDA reactions without AUDG and with AUDG were carried out by adding 1 μl of simulated carryover contamination of 1 × 10^-18^ g/μl and 1 μl of diluted DNA templates (10 ng, 10 pg, 1 pg, 100 fg, 10 fg, 1 fg, and 100 ag per microliter per microliter) in the same amplification vessel. Total mass of simulated carryover contaminants (1 × 10^-18^ g) for each reaction is approximately equivalent to a 0.2-μm-diameter aerosol droplet. In the clinical and basic laboratories, the aerosol droplet could not be completely eliminated by either high efficiency particulate air filters or fibrous pipette tip filters in the biosafety cabinets (Le Rouzic, 2006; Barhate and Ramakrishna, 2007; Wang et al., 2017a). The LoD of m-MCDA assay with AUDG and without AUDG digestion before amplification was compared to validate whether the AUDG enzyme has the ability to remove false-positive results in the m-MCDA methods.

### Specificity of m-MCDA Assay

To determine the specificity of m-MCDA assay, genomic DNA (at least 10 ng per microliters) from 28 *S. aureus* and 30 non-*S. aureus* strains are used for performing m-MCDA reactions (**Table 2**). All m-MCDA results were obtained from lateral flow biosensor (LFB) and all examinations were repeated three times.

### Examination of the Feasibility of m-MCDA Assay

In this report, we then tested the applicability of m-MCDA method using the spiked blood samples. The human blood samples were acquired from a healthy donor with the written informed consent. Our study was reviewed and approved by the ethics committee of the National Institute for Communicable Disease Control and Prevention, China CDC, according to the medical research regulations of the Ministry of Health China (Approval No. ICDC2014003).

A suspension of MRSA strain (*S. aureus* ATCC 43300) (1 × 10^8^ CFU ml^-1^) was prepared in 1 ml of phosphate-buffered saline. The suspension was used for making a serial dilution (1 × 10^7^ CFU ml^-1^, 1 × 10^6^ CFU ml^-1^, 1 × 10^5^ CFU ml^-1^, 1 × 10^4^ CFU ml^-1^, 1 × 10^3^ CFU ml^-1^, 1 × 10^2^ CFU ml^-1^, and 1 × 10^1^ CFU ml^-1^). Then, each dilution was centrifuged and re-suspended in 100 μl of blood sample (a 5-day negative blood culture). The DNA templates from spiked blood samples were extracted using DNA extraction kits (QIAamp Blood Mini Kits; Qiagen, Hilden, Germany) according to the manufacture’s instructions. The extracted genomic DNA was eluted in 100 μl of elution buffer and a volume of 1 μl of extracted templates was used for m-MCDA reactions. Non-contamination blood samples were used as NC. The experiments were carried out in duplicate to ensure reproducibility and accuracy.

## Results

### Confirmation and Analysis of nuc- and mecA-MCDA Products

**FIGURE 2:**
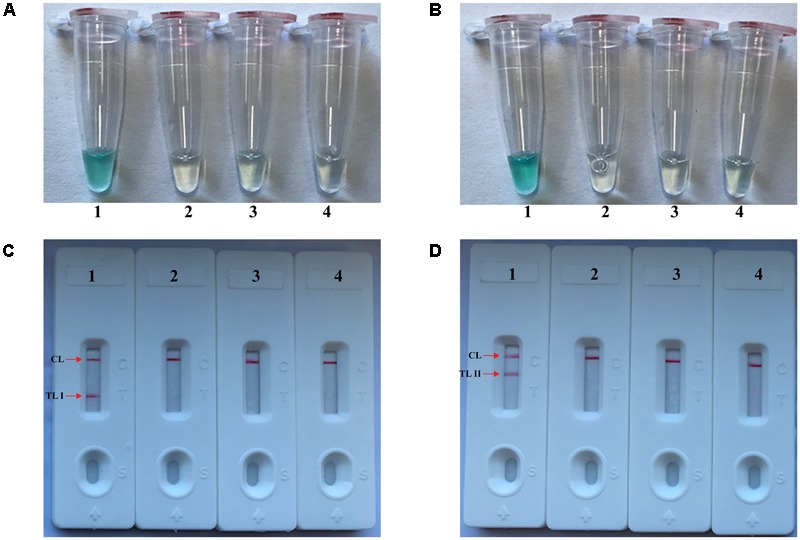
Detection and confirmation of nuc- and mecA-MCDA products. **(A,B)** Color change of nuc- and mecA-MCDA tubes; **(C,D)** LFB applied for visual detection of nuc- and mecA-MCDA products. Tube A1 (biosensor C1), positive amplification; tube A2 (biosensor C2), negative amplification (*L. monocytogenes*), tube A3 (biosensor C3), negative amplification (*S. pneumonia*), tube A4 (biosensor C4), and negative control (DW); Tube B1 (biosensor D1), positive amplification; tube B2 (biosensor D2), negative amplification (*L. monocytogenes*), tube B3 (biosensor D3), negative amplification (*S. pneumonia*), tube B4 (biosensor D4), and negative control (DW).

The monitoring techniques, including colorimetric indicator (MG) and LFB, were used for verifying and analyzing MCDA products. First, a color change of positive reactions was directly seen with unaided eyes within 1 h at a constant temperature (63°C) (**Figures 2A,B**). For *nuc* detection (**Figure 2C**), the clear crimson red bands in positive results were seen for both TL I and CL, and the TL II and CL were visible for *mecA* detection (**Figure 2D**). Moreover, only the CL appeared in the negative and blank control (**Figures 2C,D**). The results indicated that two primer sets for *nuc* and *mecA* detection were good candidates for establishment of the m-MCDA approaches.

### Optimal Amplification Temperature of the nuc- and mecA-MCDA Primer Sets

**FIGURE 3:**
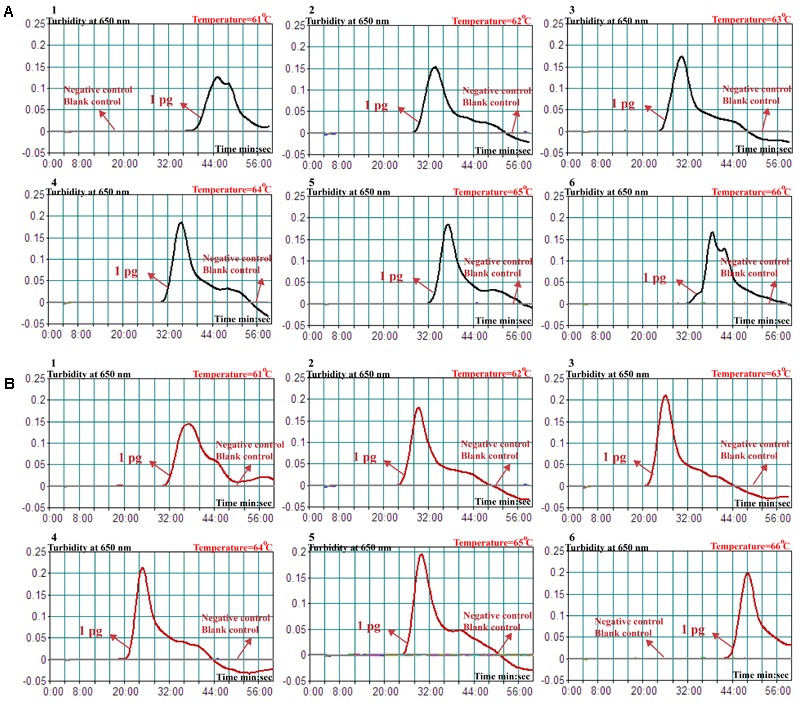
Optimal amplification temperature for nuc- and mecA-MCDA primer sets. The conventional MCDA reactions for detection of *nuc* **(A)** and *mecA* **(B)** were monitored by real-time measurement of turbidity and the corresponding curves of concentrations of templates were marked in the figures. The threshold value was 0.1 and the turbidity of > 0.1 was considered to be positive. Six kinetic graphs (1–6) were obtained at various temperatures (61–66°C, 1°C intervals) with target pathogens DNA at the level of 10 pg per vessel. A: the graphs from 2 (62°C) to 5 (65°C) showed robust amplification; B: the graphs from 2 (62°C) to 4 (64°C) showed robust amplification.

The reaction temperature plays an important role for the MCDA assay, thus the amplification temperature of nuc- and mecA-MCDA assays is optimized using genomic DNA (10 pg/μl) from purified cultures (ATCC 43300) at different reaction temperature (61–66°C) under standard MCDA protocol described above. The nuc- and mecA-MCDA reactions were analyzed by means of real-time turbidity detection, and the kinetics graphs were obtained from the all temperatures. However, the faster results were yielded for assay temperature of 62–65°C for the nuc-MCDA reactions, and 62–64°C for the mecA-MCDA reactions (**Figure 3**). The assay temperature of 63°C was used as optimal temperature for the rest of singlex and m-MCDA amplifications conducted in this report.

### Sensitivity of nuc- and mecA-MCDA Assays

**FIGURE 4:**
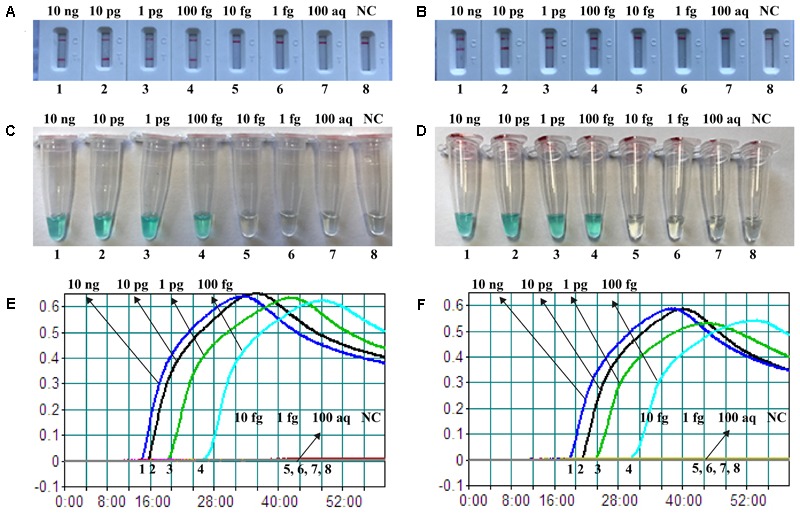
Detection of a single target in a MCDA reaction. Two sets of MCDA primers targeting the *nuc* **(A,C,E)** and *mecA* **(B,D,F)** genes were used in different reactions and the serial dilutions (10 ng, 10 pg, 1 pg, 100 fg, 10 fg, 1 fg, and 100 ag) of target templates were subjected to conventional MCDA reactions. **(A,B)** LFB applied for visual detection of *nuc*- and *mecA*-MCDA products. **(C,D)** MG applied to *nuc*- and *mecA*-MCDA products. **(E,F)** real-time turbidity applied for analysis of *nuc*- and *mecA*-MCDA products. Biosensors A1-A7 (Tubes C1–C7 and Signals E1–E7), *S. aureus* (ATCC 43300) genomic templates (10 ng–100 aq), biosensors A8 (Tube C8 and Signal E8), negative control (DW). Biosensors B1-B7 (Tubes D1–D7 and Signals F1–F7), *S. aureus* (ATCC 43300) genomic templates (10 ng–100 ag), biosensors B8 (Tube D8 and Signal F8), and negative control (DW). NC, negative control.

The sensitivity of nuc- and mecA-MCDA was determined by serially diluted genomic DNA template (10 ng, 10 pg, 1 pg, 100 fg, 10 fg, 1 fg, and 100 ag per microliter). As shown in **Figure 4**, the LoD of nuc-MCDA assay was 100 fg genomic templates per reaction, and two crimson lines (TL I, and CL) appeared on the LFB, reporting positive results for *nuc* gene. The mecA-MCDA assay was also 100 fg genomic DNA per vessel, and two crimson lines (TL II, and CL) appeared on the LFB, reporting positive results for *mecA* gene. Moreover, the LoD of MG detection (**Figures 4A,B**) for nuc- and mecA-MCDA was consistent with LFB analysis (**Figures 4C,D**) and real-time turbidity detection (**Figures 4E,F**).

### Sensitivity of m-MCDA Assay

**FIGURE 5:**
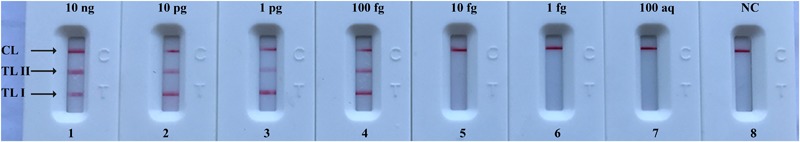
Visual detection of multiplex targets in a m-MCDA reaction. Two sets of MCDA primers targeting *nuc* and *mecA* genes were simultaneously added to a reaction vessel and the LoD of m-MCDA for simultaneously detecting *S. aureus* and identifying MRSA was analyzed using LFB. Biosensors 1, 2, 3, 4, 5, 6, 7, and 8 represent DNA levels of 10 ng (*S. aureus* ATCC 43300 templates), 10 pg (*S. aureus* ATCC 43300 templates), 1 pg (*S. aureus* ATCC 43300 templates), 100 fg (*S. aureus* ATCC 43300 templates), 10 fg (*S. aureus* ATCC 43300 templates), 1 fg (*S. aureus* ATCC 43300 templates), 100 ag (*S. aureus* ATCC 43300 templates), and negative control (DW). The LoD of m-MCDA assay for *nuc* and *mecA* detection was 100 fg per vessel.

After m-MCDA, the amplified products were directly detected using LFB. As shown in **Figure 5**, three crimson lines (TL 1, TL 2, and CL) appeared on the LFB, reporting positive results for two target genes (*nuc* and *mecA* genes). Only a crimson band (CL) appeared on the LFB, indicating negative results at the amounts lower than 10 fg of genomic DNA per vessel and blank control. The sensitivity of m-MCDA for simultaneously detecting *nuc* and *mecA* genes was also 100 fg of genomic DNA per vessels, which was in complete accordance with singlex MCDA assay (**Figures 4, 5**).

### m-MCDA Detect Simulated Carryover Contamination

**FIGURE 6:**
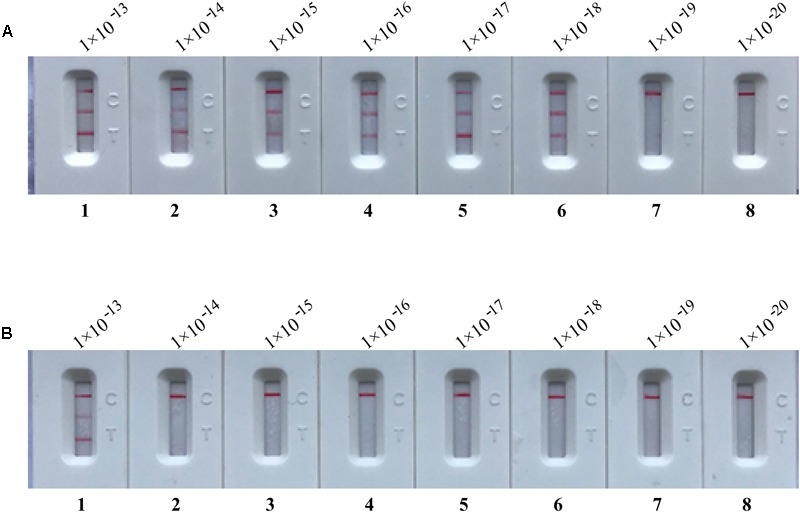
Control of carryover contamination in m-MCDA assay. Sensitivity examination of m-MCDA without AUDG treatment **(A)** and m-MCDA with AUDG treatment **(B)** using 10-fold serial dilutions of simulated carryover contamination (dUTP-incorporated products, concentration diluted from 1 × 10^-13^, 1 × 10^-14^, 1 × 10^-15^, 1 × 10^-16^, 1 × 10^-17^, 1 × 10^-18^ 1 × 10^-19^, and 1 × 10^-20^ g μL^-1^) as determined using LFB.

In order to confirm that the amplicons from m-MCDA reactions are sufficient to contaminate new m-MCDA amplifications, we then perform m-MCDA reactions with AUDG and without AUDG using serially diluted m-MCDA amplicons with concentrations ranging with 1 × 10^-13^, 1 × 10^-14^, 1 × 10^-15^, 1 × 10^-16^, 1 × 10^-17^, 1 × 10^-18^, 1 × 10^-19^, and 1 × 10^-20^ g/μL. In m-MCDA reactions without AUDG enzyme, the m-MCDA could detect as little as 1 × 10^-18^ g of simulated carryover contaminant per tube (**Figure 6A**). In m-MCDA reactions with AUDG enzyme, the m-MCDA only could detect 1 × 10^-13^ g of simulated carryover contaminant per vessel (**Figure 6B**). Our results validated that a source contaminant (1 × 10^-18^ g/μL∼0.2-μm-diameter aerosol droplet), which cannot be efficiently prevented by fibrous pipette tip filters, is sufficient to contaminate new m-MCDA reactions (**Figure 6**). However, the use of AUDG enzyme can eliminate the m-MCDA amplifications of up to 100000-fold higher concentration of carryover contaminant products, which significantly reduce the likelihood of undesired results in m-MCDA diagnosis.

### m-MCDA Assay With AUDG Enzyme Removes False-Positive Results

**FIGURE 7:**
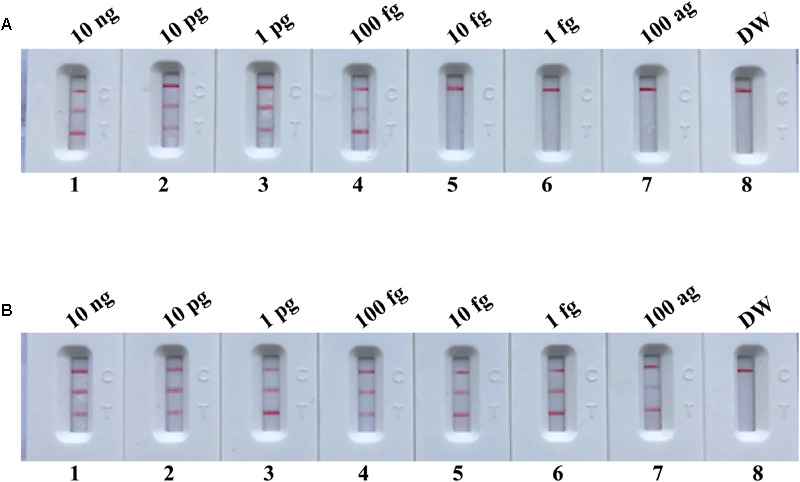
m-MCDA assay eliminates false-positive detection due to carryover contamination. Sensitivity test of m-MCDA with AUDG treatment **(A)** and m-MCDA without AUDG treatment **(B)** using serial dilutions (10 ng μl^-1^, 10 pg μl^-1^, 1 pg μl^-1^, 100 fg μl^-1^, 10 fg μl^-1^, 1 fg μl^-1^, and 100 ag μl^-1^) of ATCC 43300 and 1 × 10^-18^ g μL^-1^ of simulated carryover contamination (dUTP-incorporated m-MCDA products) as determined using LFB.

To further demonstrate that the m-MCDA method with AUDG enzyme is capable of decreasing the likelihood of unwanted results due to carryover contamination, the sensitivity determination of m-MCDA with AUDG and without AUDG enzyme is performed using serial dilution of the *S. aureus* ATCC 43300 templates. Moreover, the 1 × 10^-18^ g of contaminants (dUTP-incorporated products) also is added into each amplification vessel. In m-MCDA reactions with AUDG treatment, the LoD of m-MCDA assay is in conformity with the aforementioned sensitivity examination (**Figures 5, 7A**). In m-MCDA reactions without AUDG treatment, all evaluated samples displayed positive results, even including tested samples with undetectable level of *S. aureus* ATCC 43300 templates (less than 10 fg per vessel), which are considered as false-positive results (**Figure 7B**). As a result, we cannot correctly determine the analytical sensitivity of m-MCDA assay without AUDG enzyme treatment. These results demonstrated that the m-MCDA assay proposed here is able to successfully eliminate the unwanted results arising from carryover contamination.

### The Time Optimization of the m-MCDA Assay

**FIGURE 8:**
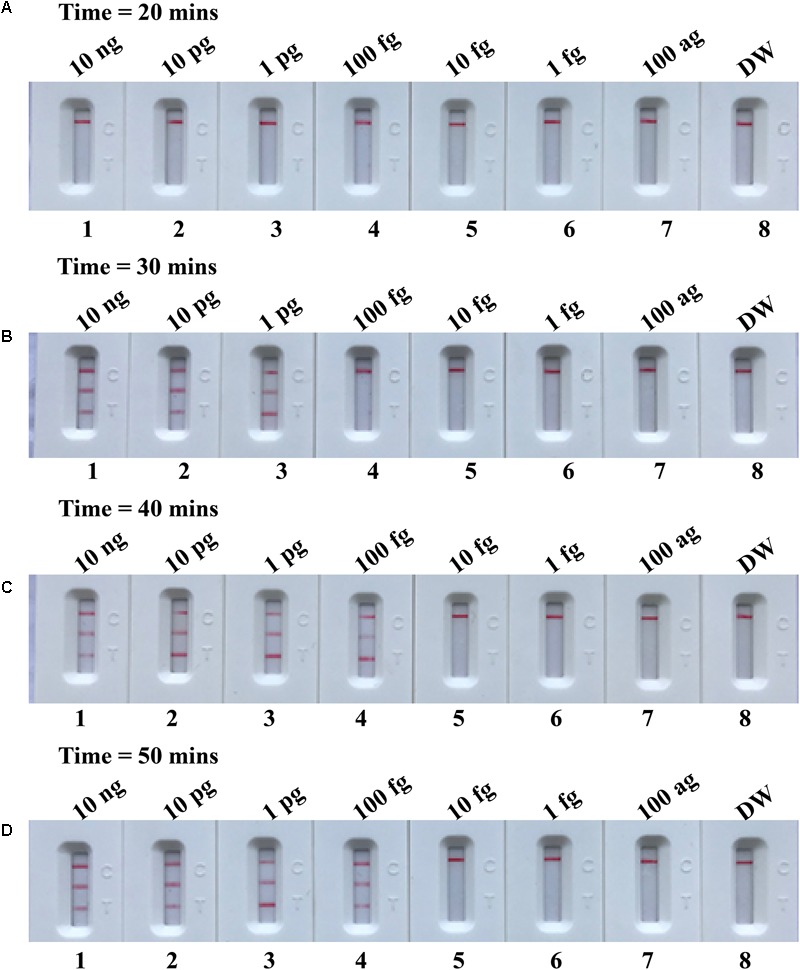
Optimal duration of time required for m-MCDA assay. Four different reaction times (**A**, 20 min; **B**, 30 min; **C**, 40 min; and **D**, 50 min) were tested and compared at 63°C. Biosensors 1, 2, 3, 4, 5, 6, 7, and 8 represent DNA levels of 10 ng μl^-1^, 10 pg, 1 pg μl^-1^, 100 fg, 10 fg μl^-1^, 1 fg μl^-1^, 100 ag μl^-1^, and blank control (DW). The best sensitivity was seen when the amplification lasted for 40 min **(C)**.

In the current report, we tested the optimal duration of time required for the m-MCDA assay during the amplification stage. A total of 4 amplification times, including 10, 20, 30, and 40 min, were compared for optimal assay time. In **Figure 8**, the lowest templates (100 fg of MRSA per reaction) displayed three crimson lines (TL 1, TL 2, and CL) when the m-MCDA reaction lasted for 40 min at 63°C. Thus, the amplification time of 40 min was used as the optimal time for the m-MCDA assay. As a result, the whole procedure, including sample processing (35 min), AUDG treatment (5 min), m-MCDA reaction (40 min), and result indicating (2 min), could be completed within 85 min.

### Analytical Specificity of m-MCDA Assay

**FIGURE 9:**
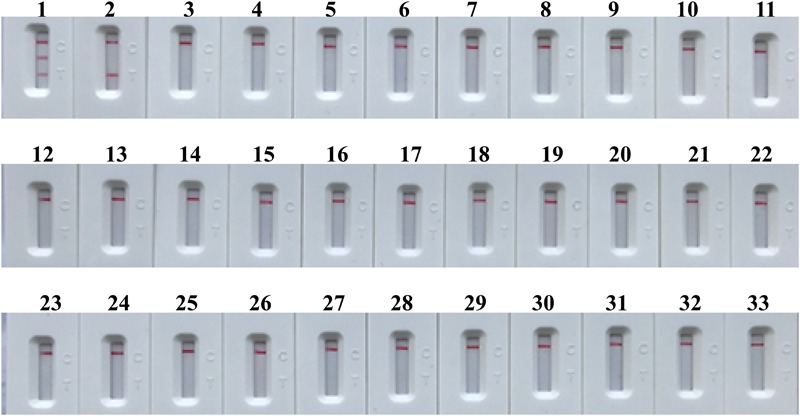
Analytical sensitivity of m-MCDA assay using different bacterial strains. The m-MCDA amplifications were performed using different genomic DNA templates and were monitored by means of visual format. Biosensor 1, MRSA (ATCC 43300); biosensor 2, MSSA (ATCC 25923); biosensor 3–32, *Vibrio cholerae, Campylobacter jejuni, Pseudomonas aeruginosa, Staphylococcus epidermidis, Vibrio alginolyticus, Plesiomonas shigelloides, Aeromonas hydrophila, Enterohemorrhagic E. coli, Enteropathogenic E. coli, Enterotoxigenic E. coli, Enteroaggregative E. coli, Enteroinvasive E. coli, Shigella dysenteriae, Shigella boydii, Shigella flexneria, Shigella sonneri, Salmonella, Enterococcus faecalis, Enterococcus faecium, Listeria monocytogenes, Listeria ivanovii, Listeria grayi, Listeria innocua, Listeria welshimeri, Listeria seeligeri, Yersinia enterocolitica, Enterobacter cloacae, Streptococcus pneumonia, Bacillus cereus, Klebsiella pneumoniae*; biosensor 33, and negative control (DW).

The specificity of m-MCDA assay is determined using extracted templates from MRSA strains, MSSA strains, and non-*S. aureus* strains. After a 40-min amplification at 63°C, positive results were generated only with the genomic DNA templates extracted from *S. aureus* (MRSA and MSSA) (**Figure 9** and **Table 2**). Three crimson lines, including TL 1, TL 2, and CL, appeared on the LFB, reporting the positive results for MRSA strains **(Figure 9**, biosensor 1), whereas TL 1 and CL appeared on the biosensor, reporting the positive results for MSSA strains (**Figure 9**, biosensor 2). Herein, the m-MCDA assay established here could detect all *S. aureus* stains, and differentiate MRSA from MSSA. Moreover, the analytical specificity of m-MCDA assay was of 100% (**Table 1**), and no cross-reactions to non-*S. aureus* strains were produced according to the specificity testing. Our results verified that the m-MCDA technique exhibited high specificity for analysis of *S. aureus* strains.

### Applicability of m-MCDA Assay to Blood Samples

**FIGURE 10:**
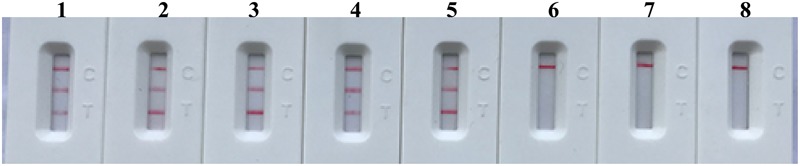
Analytical sensitivity of m-MCDA for detecting target sequence in blood samples. The monitoring technique (LFB) was applied for analyzing the m-MCDA amplification products. The serial dilutions of target templates (ATCC 43300) were subjected to m-MCDA reactions. Strips (1–8) represented the DNA levels of 100000, 10000, 1000, 100, 10, 1, 0.1 CFU per reaction, and negative control (non-contaminated blood samples). The genomic DNA levels of 100000, 10000, 1000, 100, and 10 CFU per reaction produced the positive reactions.

To further demonstrate the feasibility of m-MCDA as a valuable tool for *S. aureus* analysis, we analyzed the artificially contaminated blood samples with MRSA strain (ATCC 43300) by using m-MCDA assay. The m-MCDA method yielded the positive signals when the contaminated numbers of MRSA (ATCC 43300) were more than 1 × 10^3^ CFU ml^-1^ (10 CFU per reaction), and the *nuc* and *mecA* genes were simultaneously detected (**Figure 10**). The m-MCDA assay produced the negative results when the contaminated numbers of MRSA (ATCC 4330) were less than 1 × 10^2^ CFU ml^-1^ (1 CFU per reaction). Moreover, no positive results were observed in NCs (non-spiked blood samples).

## Discussion

*Staphylococcus aureus*, including MRSA (methicillin-resistant *S. aureus*), is an important bacterium that produces various toxins, and is responsible for a variety of infections, including bacteremia, soft-tissue infections, and staphylococcal food poisoning (Udo et al., 2009; Pai et al., 2010; Hanberger et al., 2011). The prompt detection of *S. aureus* from various samples and identification of methicillin resistance is essential in cases of suspected infections. However, the conventional detection techniques, including culture-based methods, colony morphology, micro-dilution resistance examinations, and PCR-based assays (traditional PCR methods and real time PCR approaches), are time-consuming and laborious (Shrestha et al., 2002). Hence, a simple, rapid, reliable assay is required for application in a basic laboratory or a hospital clinical laboratory.

In order to obtain more such effective detection tool, an m-MCDA assay targeting *nuc* and *mecA* genes was successfully developed for detecting all *S. aureus* strains and differentiating MRSA from MSSA. In the MCDA-based assay, MCDA primer set, which specially binds 10 regions of the target genes, provides a high degree of selectivity (**Figure 1**). The sequences of nuc-MCDA primer set were designed using the species-specific gene (*nuc* gene), which is unique to all *S. aureus* strains. Moreover, methicillin-resistance in *S. aureus* is primarily mediated by the low-affinity penicillin-binding protein 2a or 2′ (PBP2a or PBP2′), which is encoded by the chromosomal structural gene *mecA*. Herein, the sequences of mecA-MCDA primer set are designed using the *mecA* gene, which is associated with methicillin-resistance of *S. aureus* strains (Hanaki et al., 2011). The assay’s specificity was determined with the genomic templates extracted from 28 *S. aureus* (15 MRSA and 13 MSSA) and 30 non-*S. aureus* strains, and the positive results were yielded from the assay of all *S. aureus* strains but not for non-*S. aureus* strains. The m-MCDA assay targeting the *nuc* gene identified *S. aureus* with 100% specificity, and m-MCDA assay targeting the *mecA* gene associated with methicillin resistance identified methicillin-resistant *S. aureus* with 100% specificity (**Figure 9** and **Table 2**). Importantly, the m-MCDA method developed here could detect all *S. aureus* stains, and distinguish MRSA from MSSA.

In the current study, we applied the LFB to analyze MCDA products, because of its simple operation, rapid results, and ease of use in clinical laboratory and field settings. Analysis of MCDA amplicons with LFB is not only fast, but also simpler and less error-prone than analysis by the other monitoring techniques (such as real time turbidity and colorimetric indicator) employed in the present study (**Figures 2, 3**). Due to elimination of the use of special reagent, instrument and process, LFB was more suitable than other analysis methods for rapid, simple and sensitive detection of MCDA products. Most importantly, the LFB used in our report can simultaneous and visual detection of two targets in a single test (Wang et al., 2017c). The MCDA assays for independently identifying *nuc* and *mecA* genes were 100 fg of DNA templates per tube, the LoD of LFB detection for MCDA products was conformity with real-time turbidity analysis and colorimetric indicator (MG) detection (**Figure 4**). The LoD of m-MCDA assays for simultaneously detecting *nuc* and *mecA* genes were also 100 fg of genomic templates per vessel, which was consistent with the singlex nuc-MCDA and mecA-MCDA detection (**Figures 4, 5**). For spiked blood samples, m-MCDA assay produce the positive signals when the contaminated numbers of MRSA (ATCC 43300) were more than 1 × 10^3^ CFU ml^-1^ (10 CFU per reaction), and the *nuc* and *mecA* genes were simultaneously detected. Moreover, the MCDA amplification can be carried out with only simple equipment (such as a regular heat block or laboratory bath) that offers a constant temperature of 63°C, avoiding the use of complex instrument.

Opening of the amplification tube is an essential step for reporting the MCDA result by LFB, which can produce aerosol droplets of different sizes that contain high concentration of MCDA products. Due to its high sensitivity, the MCDA amplicons generated from previous reactions is one of very tricky problems because it can yield undesired results (Wang et al., 2017a). Our results demonstrated that a trace amount of contaminants (1 × 10^-18^ g/vessel) can produce false-positive results, thus removing carryover contamination is a pivotal factor for accurate and reliable MCDA detection. Here, our study successfully prevented the carryover contamination using two additional components (AUDG enzyme and dUTP). Firstly, dUTP was incorporated instead of dTTP into all MCDA amplicons. Next, prior to MCDA amplification, we treat the MCDA mixture with AUDG enzyme at room temperature for only 5 min, thus the amplified DNA from previous reaction could be specifically digested by AUDG enzyme (Wang et al., 2017b). The target templates, which are uracil-free DNA, remain completely unaffected (Kil et al., 2015). The AUDG is a heat-labile enzyme, and is automatically and rapidly deactivated when MCDA is performed at an elevated temperature (i.e., 63°C). As a result, the use of the AUDG enzyme enables the MCDA method to be performed in a single closed tube (Wang et al., 2017b). Genuine MCDA amplicons subsequently produced from the target templates during the MCDA reaction were not cleaved, allowing MCDA amplification to proceed normally. Thus, entire procedure, including sample processing (35 min), AUDG treatment (5 min), m-MCDA reaction (40 min), and result indicating (2 min), could be completed within 80 min (**Figure 8**).

## Conclusion

An m-MCDA assay for simultaneous detection of *S. aureus* strains and identification of MRSA based on *nuc* and *mecA* gene was successfully developed. This approach established in the present study exhibited high specificity for target template analysis, and had the analytical sensitivity of 100 fg per vessel with pure culture and 10 CFUs per tube with spiked blood samples. The reaction products were analyzed using LFB, which was objective, easy-to-use, and disposable. Moreover, the false-positive results arising from carryover contamination could be eliminated by using AUDG enzyme and dUTP. Herein, the m-MCDA assay developed here was a simple, rapid, sensitive, and reliable technique to detect all *S. aureus* strains, and identify MRSA infection for appropriate antibiotic therapy.

## Author Contributions

YiW, JX, and CY conceived and designed the experiments. YiW, WY, SF, and YaW performed the experiments. YiW analyzed the data. YiW, WY, SF, SH, and YaW contributed the reagents, materials, and analysis tools. YiW performed the software. YiW, JX, and CY wrote the paper.

## Conflict of Interest Statement

The authors declare that the research was conducted in the absence of any commercial or financial relationships that could be construed as a potential conflict of interest.
